# Novel biomarker *ZCCHC13* revealed by integrating DNA methylation and mRNA expression data in non-obstructive azoospermia

**DOI:** 10.1038/s41420-018-0033-x

**Published:** 2018-02-26

**Authors:** Zhiming Li, Shuai Chen, Yufeng Yang, Xuan Zhuang, Chi-Meng Tzeng

**Affiliations:** 1 Translational Medicine Research Center—Key Laboratory for Cancer T-Cell Theranostics and Clinical Translation, School of Pharmaceutical Sciences, Xiamen University, Xiamen, Fujian China; 2 Department of Urology, The First Affiliated Hospital of Xiamen University, Xiamen, Fujian China; 3 Department of Urology, The First Clinical Medical College of Fujian Medical University, Fuzhou, Fujian China; 4 Institute of Translational Medicine, Nanjing Medical University, Nanjing, Jiangsu China; 5 College of Pharmaceutical Sciences, Nanjing Tech University, Nanjing, Jiangsu China

## Abstract

The objective of this study was to identify genes regulated by methylation that were involved in spermatogenesis failure in non-obstructive azoospermia (NOA). Testis biopsies of patients with NOA and OA (with normal spermatogenesis) were evaluated by microarray analysis to examine DNA methylation and mRNA expression using our established integrative approach. Of the coordinately hypermethylated and down-regulated gene list, zinc-finger CCHC-type containing 13 (*ZCCHC13*) was present within the nuclei of germ cells of testicular tissues according immunohistochemistry, and there was decreased protein expression in men with NOA compared with OA controls. Mechanistic analyses indicated that ZCCHC13 increased c-MYC expression through the p-AKT and p-ERK pathways. To confirm the changes in *ZCCHC13* expression in response to methylation, 5-aza-2′-deoxycitidine (5-Aza), a hypomethylating agent, was administered to mouse spermatogonia GC-1 cells. We demonstrated that 5-Aza enhanced protein and mRNA expression of *ZCCHC13* epigenetically, which was accompanied by activation of p-AKT and p-ERK signaling. Our data, for the first time, demonstrate that ZCCHC13 is an important signaling molecule that positively regulates the AKT/MAPK/c-MYC pathway and that methylation aberrations of *ZCCHC13* may cause defects in testis development in human disease, such as NOA.

## Introduction

Azoospermia is typically classified as either obstructive azoospermia (OA) or non-obstructive azoospermia (NOA). The presence of OA is mainly due to a physical obstruction of the post-testicular genital tract, but there is normal spermatogenesis in more than 90% of cases^1^. However, NOA is defined as the absence of spermatozoa in the ejaculate because of a spermatogenesis dysfunction, which is the most serious type of male infertility. The majority of NOA diagnoses are idiopathic and the etiology is mostly unknown, although abnormal karyotype and chromosome Yq microdeletions are observed in NOA patients. It has been reported that men with NOA account for nearly 10% of all infertile males^2^. Nevertheless, NOA patients have the potential to father a child even with very low production of sperm if the sperm can be successfully retrieved by intracytoplasmic sperm injection and used for in vitro fertilization. However, andrologists still face problems with retrieved gametes despite the fact that the gametes are fertile, such as the uncertainty of the reproductive probability and risk of birth defects in pregnancies. Testicular biopsy may destroy the focal spermatogenetic areas for future retrieval attempts, even though, currently, it is the “gold-standard” diagnostic test for NOA. Hence, further investigation is desirable in NOA patients to better understand the mechanisms of spermatogenetic failure and identify the biomarkers underlying NOA.

To date, 21–39% of azoospermia cases can be explained by genetic defects. These cases have a molecular diagnosis based on multiplex PCR analysis that has been used to detect NOA cases^3^. However, most cases of azoospermia are idiopathic and unrelated to known genetic factors. Epigenetic studies have recently shown potential etiologies of DNA methylation for the failure of spermatogenesis, including aberrant modification of both imprinted genes (*MEST*, *GNAS*, and *H19*) and nonimprinted genes (*P16*, *MTHFR*, *CREM*, and *DAZL*)^4,5^. DNA methylation is a well-studied epigenetic mechanism that alters the level of mRNA expression without any changes in the DNA sequence. Several studies have shown that there are aberrant DNA methylation patterns in spermatozoa in men with unexplained infertility^6,7^. The emergence of microarray and sequence technologies allows for the analysis of alterations of DNA methylation at the whole-genome level. Moreover, a number of integrative studies have recently defined molecular profiles for some common diseases (including Crohn’s disease^8^, lung adenocarcinoma^9^, obesity^10^, and rheumatoid arthritis^11^). The lack of discoidin domain receptor 1 (*DDR1*) gene expression in NOA patients has been revealed by integrative analysis of DNA methylation and gene expression^12^. DDR1 is a receptor tyrosine kinase expressed in human postmeiotic germ cells and is associated with cell growth, apoptosis, morphogenesis, and differentiation^13^. We propose that misregulation of methylation patterns at other genes involved in the pathophysiology of male infertility can be identified through these integrative methods.

While previous studies have profiled DNA methylation in azoospermia, their analyses have either been limited by the semen samples, which are not sufficient to analyze due to complex alterations in testis development, or by the lack of gene expression analysis, which allows the potential effects of DNA methylation changes to be illustrated. Recently, we have reported an integrative method to identify significant genes that are dysregulated by DNA methylation modifications in cases of severe oligospermia^14^. The aim of this study is to identify the novel biomarkers and gain insight into the molecular mechanisms of idiopathic NOA using our published integrative analysis of DNA methylation and mRNA expression profiles. First, we analyzed the DNA methylation and mRNA expression profiles in four NOA and three age-matched OA testicular tissues with normal spermatogenesis using the high-resolution Infinium 450K Methylation Array and Agilent SurePrint G3 Human Gene Expression 8 × 60K Array. Of the hypermethylated and down-regulated genes identified from the integrated analysis, zinc-finger CCHC-type containing 13 (*ZCCHC13*) had lower mRNA expression levels in men with NOA compared with normozoospermic OA individuals. From the top Gene Ontology (GO) terms and Kyoto Encyclopedia of Genes and Genomes (KEGG) categories, we found that aberrant cell cycle and mitogen-activated protein kinase (MAPK) signaling pathways were associated with NOA in our functional analysis. Furthermore, we identified that ZCCHC13 expression positively correlated with the phosphorylation levels of AKT and ERK and expression level of c-MYC. We also observed that 5-Aza-2′-deoxycitidine (5-Aza)-induced demethylation epigenetically increased the expression of *ZCCHC13*. Taken together, these results for the first time reveal the patterns of methylation and transcription alterations in NOA and provide direct molecular evidence for etiologies of azoospermia.

## Results

### Comparative analysis of global DNA methylation patterns between NOA and OA

**Fig. 1:**
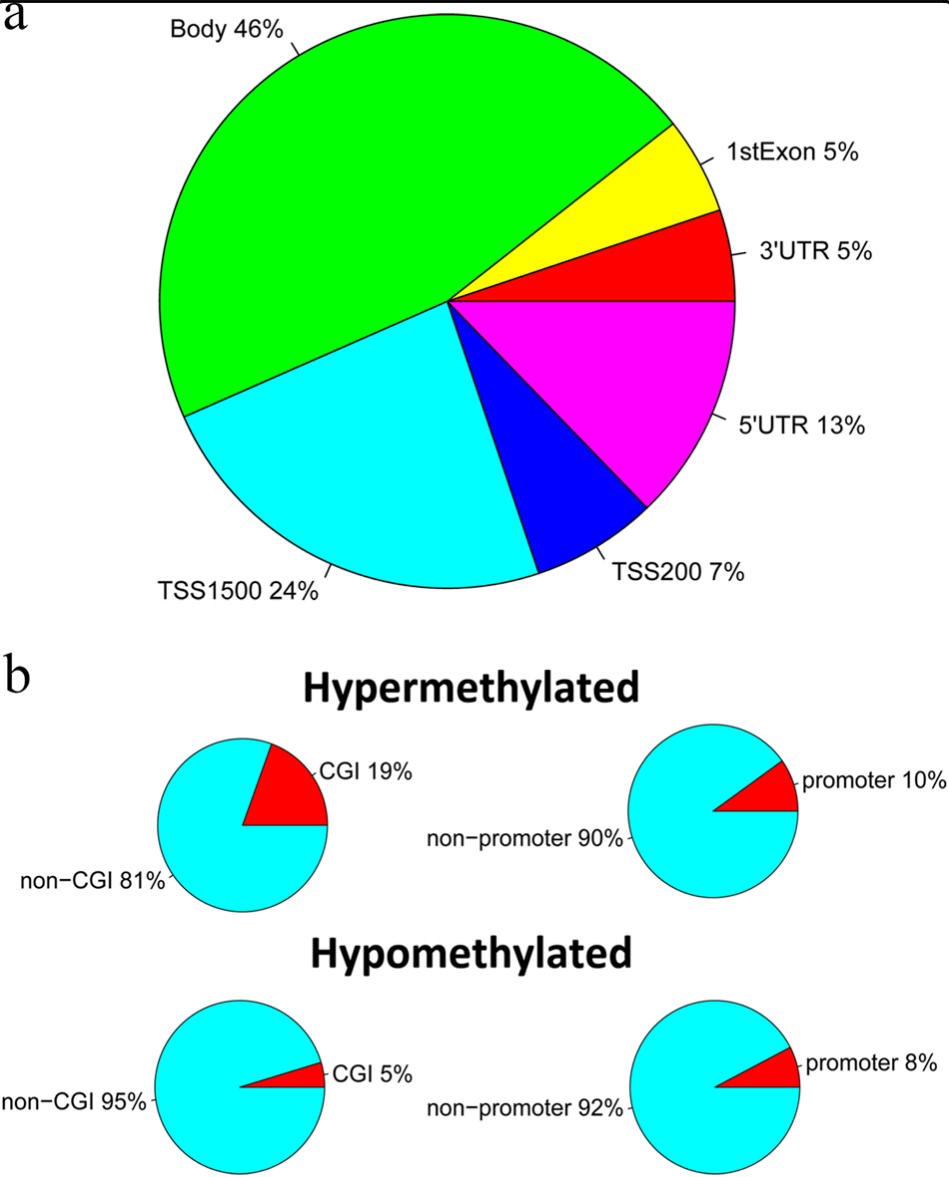
Identification of DNA methylation differences between NOA and OA. **a** Genomic distribution of differentially methylated probes regarding their respective location to genes. **b** Proportions of hypermethylated and hypomethylated probes from genes with associated CpG islands (CGIs) and probe locations, categorized as promoter ( ± 1 kb from TSS) or non-promoter regions

High-throughput DNA screening was used to compare the methylation patterns of testis biopsies of NOA patients and OA patients. The methylation bead array allowed the interrogation of >450,000 CpG sites across the entire genome, covering 99% of RefSeq genes. Statistical analysis in our study showed that 30,697 CpG sites, associated with 10,174 different genes, had significant differences in DNA methylation between NOAs and OAs (absolute value of Δ*β* > 0.20, *p* < 0.05). Specifically, we found 10,600 hypomethylated CpG sites in 4,253 genes and 20,097 hypermethylated CpG sites in 7,889 genes. Some of our most hypermethyalted loci, such as *MEST* and *DAZL*, passed our strict cutoffs, supporting previous observations of abnormal DNA methylation of imprinted and nonimprinted genes in male infertility^15–17^. The differentially methylated CpGs sites were distributed throughout the genome; 46% of sites were located in a gene body, 24% of sites were located 1,500 bp from the transcription start site (TSS1500), 13% of sites were located in the 5′-untranslated region (UTR) region, 7% of sites were located 200 bp from the transcription start site (TSS200), and 5% of sites were located in the 3′-UTR region or the first exon (Fig. 1a). For the differentially methylated probes, we also analyzed the CpG island (CGIs) distribution and promoter distribution (defined as the region 1 kb upstream or downstream of the nearest transcription start site), respectively (Fig. 1b). Our data showed that 19% of CpGs in CGIs and 81% of CpGs in non-CGIs were hypermethylated, while 5% of CpGs in CGIs and 95% of CpGs in non-CGIs were hypomethylated. On the other hand, 10% of CpGs in promoters and 90% of CpGs in non-promoters were hypermethylated, while 8% of CpGs in CGI and 92% of CpGs in non-CGI were hypomethylated. It is noteworthy that the hypermethylated loci included the *CDK* gene family, including *CDK4*,* CDK5*,* CDK6*,* CDK18*,* CDK19*, and* CDK20*. These genes are involved in modulation of the cell cycle during spermatogenesis, which is required to ensure self-renewal of male germline cells and differentiation of appropriate numbers of cells for the various lineages^18^. Our findings supported a recent observation that the *DDR1* promoter was hypermethylated in NOA patients^12^. Additionally, the remarkable hypomethylations of *CECR2*, *GRAP2*, *CCL23*, and *SLA2* in NOA patients were in accordance with previous microarray data^12^. Our differentially methylated gene analysis consisted of some genes with known implications for male infertility pathogenesis and also contained many potentially interesting novel genes. For the latter group, little or nothing is known about the DNA methylation-based deregulation of these genes in spermatogenesis.

### Integrated analysis of DNA methylation and mRNA expression

**Fig. 2:**
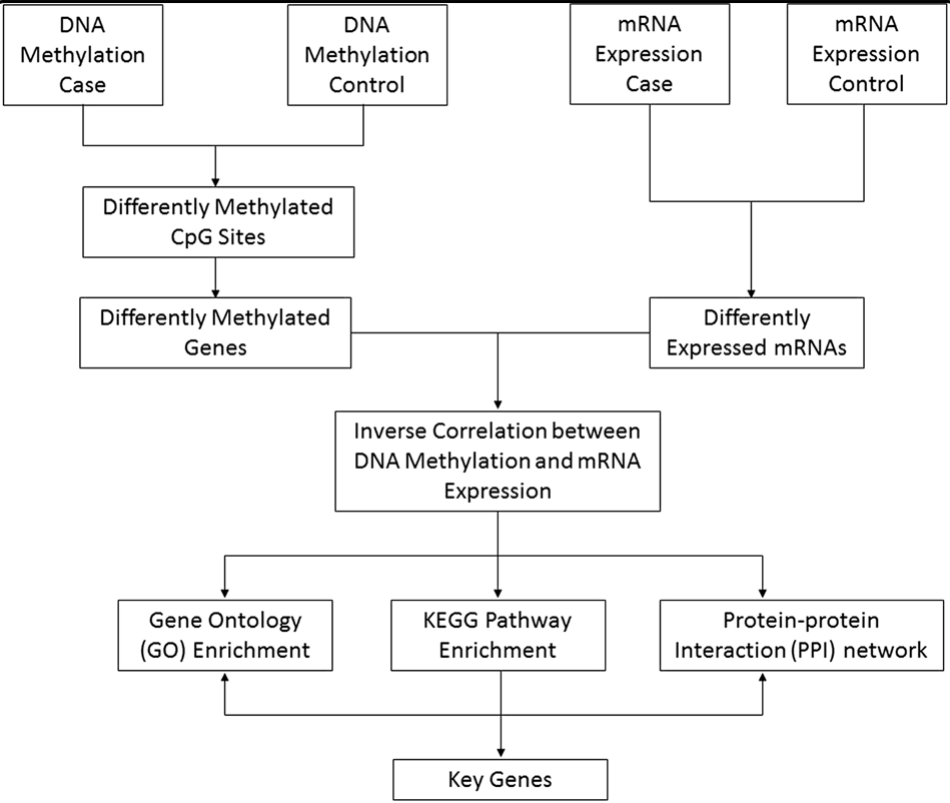
Schematic pipeline depicting the strategy of identifying key genes from DNA methylation and mRNA expression microarray data. The method proceeds as follows:(1) identify significantly differentially methylated genes and expressed genes in the case and control groups; (2) retain those genes whose methylation and expression levels are highly anti-correlated; (3) enrich the gene ontology, KEGG pathway, and PPI networks of anti-correlated genes; (4) identify key genes from the intersection of statistical analysis, gene functional annotations, and interaction networks

**Fig. 3:**
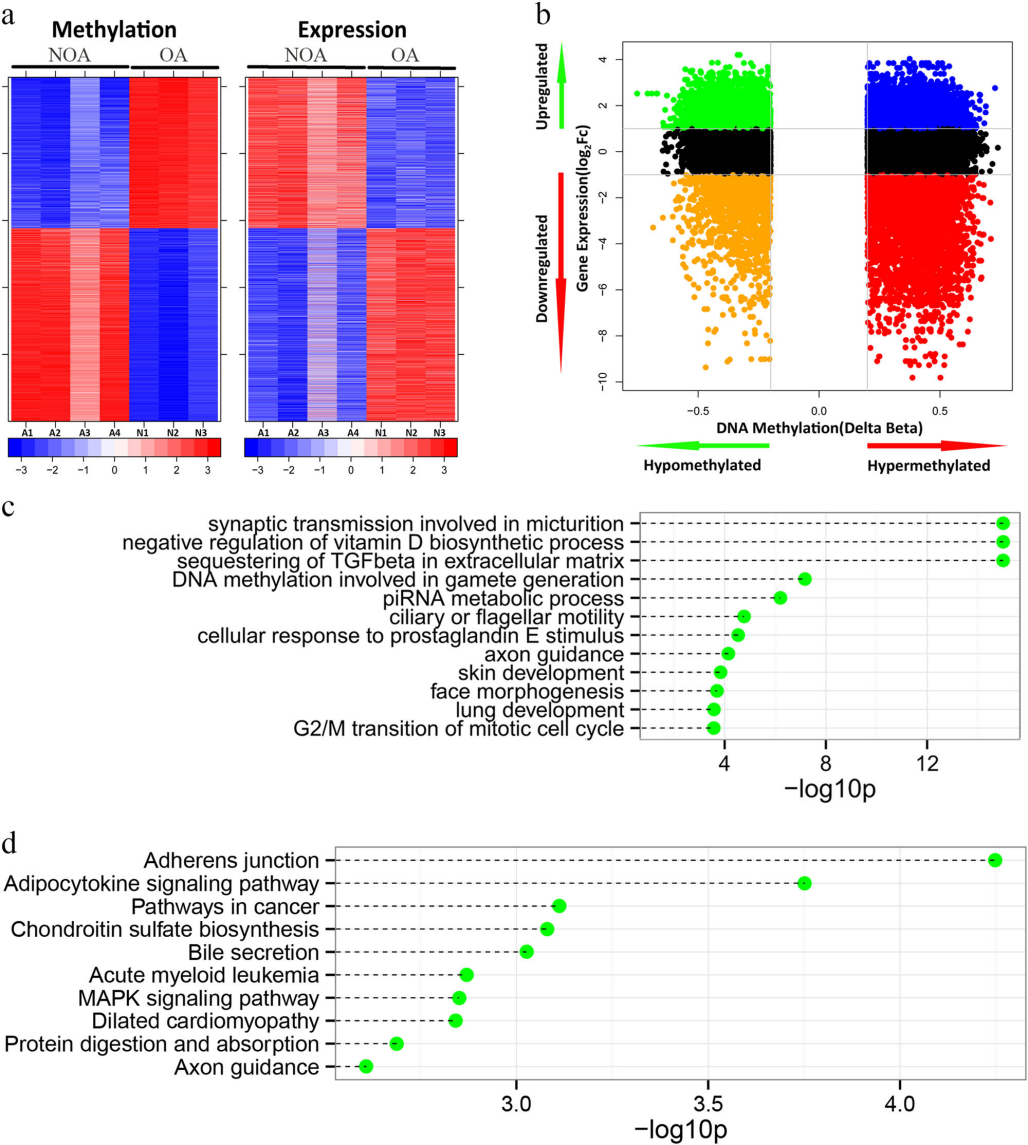
Integration of DNA methylation with expression data. **a** Heatmap comparison of inversely correlated expression and methylation. **b** Starburst plot integrating differential DNA methylation and gene expression analysis. Indicated are genes that are hypermethylated and down-regulated genes (red); hypomethylated and up-regulated genes (green); hypermethylated and up-regulated genes (blue); or hypomethylated and down-regulated genes (orange). **c** The top 12 most significantly enriched biological process categories and zinc-finger CCHC-type containing 13 (the top10 most significantly enriched KEGG pathways within genes showing significant DNA methylation changes associated with significant inverse gene expression changes

**Fig. 4:**
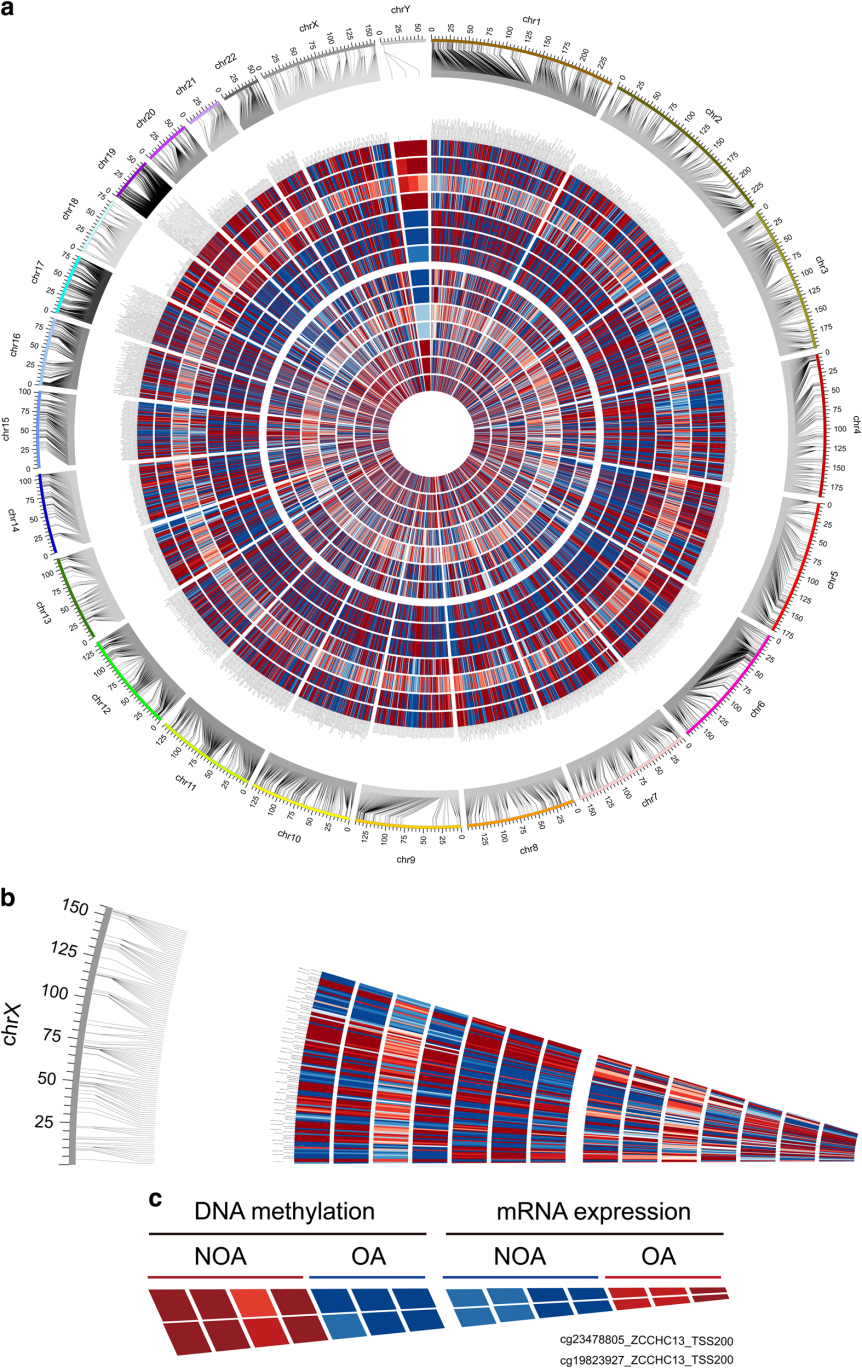
Integrated Circos plot of genes showing coordinately inversed DNA methylation and gene expression. The outermost circle displays the human chromosomes. The inner six circles (four NOA and three OA samples displayed from outside to inside) represent the genome-wide distribution of differentially methylated probes. The innermost circles (four NOA and three OA samples displayed from outside to inside) represent the globally differentially expressed genes. The red indicates hypermethylation or overexpression, and blue indicates hypomethylation or under-expression

To identify DNA methylation changes with concomitant changes in gene expression, we integrated the gene expression profiles and DNA methylation profiles of the NOAs and OAs. Differentially methylated genes between the two groups were identified and combined with data from gene expression profiles using our generated integrative approach (Fig. 2)^19^. It is well established that changes in DNA methylation and differences in gene regulation are causally related, with hypomethylation generally leading to gene expression and hypermethylation resulting in gene silencing. Based on the calculated correlations between methylation and expression data, the highly anti-correlated genes were retained and analyzed in the following steps. A total of 2,597 genes with 5,138 methylated probes were significantly inversely correlated with changes in expression (*p* < 0.05, *ρ* < 0). Both DNA methylation profiles and mRNA expression profiles based on unsupervised hierarchical clustering identified two unique clusters that had distinct signatures (Fig. 3a). Table 1 shows the most significant 15 genes with a negative correlation between DNA methylation and mRNA expression in NOA vs. OA. The chromosome distribution of these regions was then analyzed: 5,138 methylated probes of 2,597 genes were located to 24 human chromosomes (Fig. 4a). Recent studies have frequently detected altered copy number variants and mutations of X-chromosome genes in patients with a failure of spermatogenesis^20,21^. In our data, genes with aberrant methylation modifications on the chromosome X were also observed (Fig. 4b). One X-chromosome gene, *ZCCHC13*, had decreased methylation associated with increased expression in NOA (Fig. 4c). In total, 1,164 genes were statistically significantly hypermethylated and down-regulated (44.8%), while 834 genes (32.1%) were significantly hypomethylated and up-regulated (Fig. 3b). In addition to those genes that had relationships between DNA methylation and gene expression changes, we observed some genes that had a positive association, which were hypermethylated but up-regulated or hypomethylated and down-regulated. Therefore, we found both negative and positive associations between DNA methylation and gene expression, which may imply the existence of two different mechanisms of DNA methylation-dependent gene regulation for NOA.

**Table 1:** Top 15 genes with the negative correlation of DNA methylation and mRNA expression between NOA and OA

Probe name	Gene symbol	Gene ID	Reference accession	Region	*ρ* value	*P* value methylation	*Q* value methylation	Δ*β* value (NOA/OA)	Fold change (NOA/OA)
cg07085664	*CHRNB3*	1142	NM_000749	Body	−0.757	0.048	0.124	0.442	0.429
cg07421329	*EFHA2*	286097	NM_181723	Body	−0.757	0.048	0.124	−0.345	6.455
cg02134046	*SPERT*	220082	NM_152719	3′-UTR	−0.786	0.034	0.100	0.413	0.002
cg13823366	*FBXO39*	162517	NM_153230	TSS1500	−0.786	0.034	0.100	0.387	0.003
cg00601486	*H1FNT*	341567	NM_181788	1st Exon	−0.786	0.034	0.100	0.504	0.005
cg24153003	*OAZ3*	51686	NM_001134939	Body	−0.786	0.034	0.100	0.213	0.005
cg27623334	*CXorf61*	203413	NM_001017978	TSS200	−0.786	0.034	0.100	0.544	0.006
cg13513349	*ADAM29*	11086	NM_001130704	5′-UTR	−0.786	0.034	0.100	0.445	0.007
cg15822411	*CCDC83*	220047	NM_173556	1st Exon	−0.786	0.034	0.100	0.310	0.009
cg09848324	*PDCL2*	132954	NM_152401	Body	−0.786	0.034	0.100	0.514	0.009
cg26647524	*KBTBD5*	131377	NM_152393	TSS200	−0.786	0.034	0.100	0.418	0.010
**cg23478805**	***ZCCHC13***	**389874**	**NM_203303**	**TSS200**	−**0.786**	**0.034**	**0.100**	**0.572**	**0.010**
cg22806002	*BTG4*	54766	NM_017589	TSS200	−0.786	0.034	0.100	0.218	0.011
cg04864648	*PDHA2*	5161	NM_005390	TSS200	−0.786	0.034	0.100	0.492	0.012
cg24803912	*POU5F2*	134187	NM_153216	TSS200	−0.786	0.034	0.100	0.465	0.012

*NOA* non-obstructive azoospermia, *OA* obstructive azoospermia

Bold values indicated that DNA methylation and mRNA expression of ZCCHC13 was highly anticorrelated and was ranked very top

### Identification of DNA methylation-derived biological changes in NOA

From the standpoint of the classical paradigm of DNA methylation regulation, genes with reciprocal changes in expression and methylation were identified. We then set out to determine the biological dysfunctions caused by the inversely correlated genes for NOA pathogenesis. We therefore performed GO analysis to test which biological processes were significantly associated with NOA. The analysis was performed for genes with both hypomethylated and hypermethylated alterations. We observed a number of significantly enriched functional processes that are potentially relevant in the biology of NOA (Fig. 3c) that included the following categories: synaptic transmission involved in micturition, negative regulation of the vitamin D biosynthetic process, sequestering of tumor growth factor-β in the extracellular matrix, DNA methylation involved in gamete generation, Piwi-interacting RNA (piRNA) metabolic process, ciliary or flagellar motility, cellular response to prostaglandin E stimulus, axon guidance, skin development, face morphogenesis, lung development, and G2/M transition of mitotic cell cycle. Enriched biological processes include functional regulation of vitamin D, vitamin E, and piRNA and structural assembly capabilities for spermatogenesis, which are well known to be involved in male reproduction health. These results suggested that DNA methylation is potentially implicated in a series of events that are important for spermatogenesis.

**Fig. 5:**
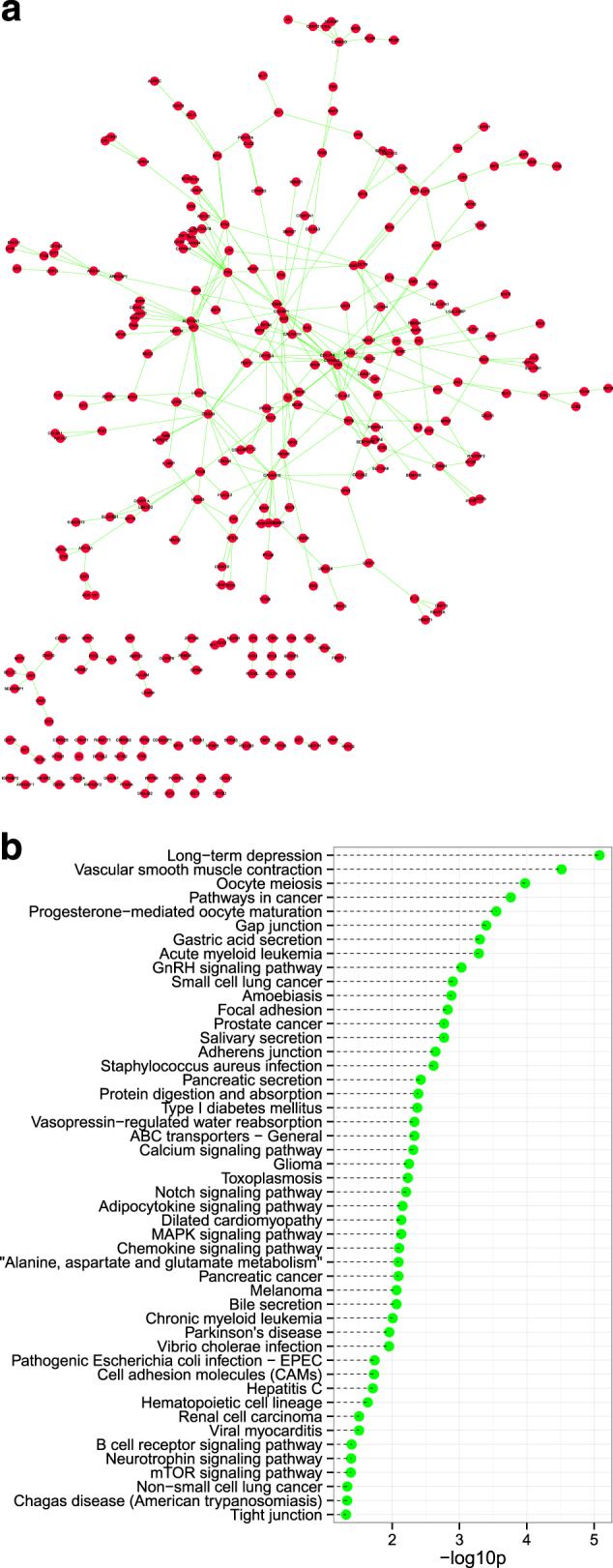
Using PPI to infer core elements involved in NOA. **a** Protein–protein interaction network for genes differentially expressed and DNA methylated in NOA. The red circles represent the hub genes with inversely correlated DNA methylation and gene expression. **b** KEGG pathway analysis of the PPI network. The *p*values were calculated using hypergeometric tests and corrected using the Benjamini–Hochberg adjustment. The corrected *p*values are expressed and presented as negative logarithms (base 10)

To identify the most relevant cellular activities controlled by these inversely correlated genes, we performed the KEGG pathway analysis. The most significant pathways were related to adherens junction, the adipocytokine signaling pathway, cancer pathways, chondroitin sulfate biosynthesis, bile secretion, acute myeloid leukemia, the MAPK signaling pathway, dilated cardiomyopathy, protein digestion and absorption, and axon guidance (Fig. 3d). Furthermore, to find possible associations within negatively correlated genes, we created a protein–protein interaction network consisting of 286 protein nodes and 337 interactions based on the inversely correlated genes using the Human Protein Reference Database (Fig. 5a). We also observed the MAPK signaling pathway in the KEGG analysis of PPI networks (Fig. 5b). The MAPK signaling pathway is a conserved signaling cascade that utilizes a series of kinases to transduce signals from the cell membrane to the nucleus, thereby mediating cell growth, cell survival and cell differentiation. To delineate molecular mechanisms underlying DNA methylation modification, we identified a potential gene, *ZCCHC13*, that showed remarkably significant changes in DNA methylation and gene expression in the microarray data.

### ZCCHC13 expression is associated with patients with NOA

**Fig. 6:**
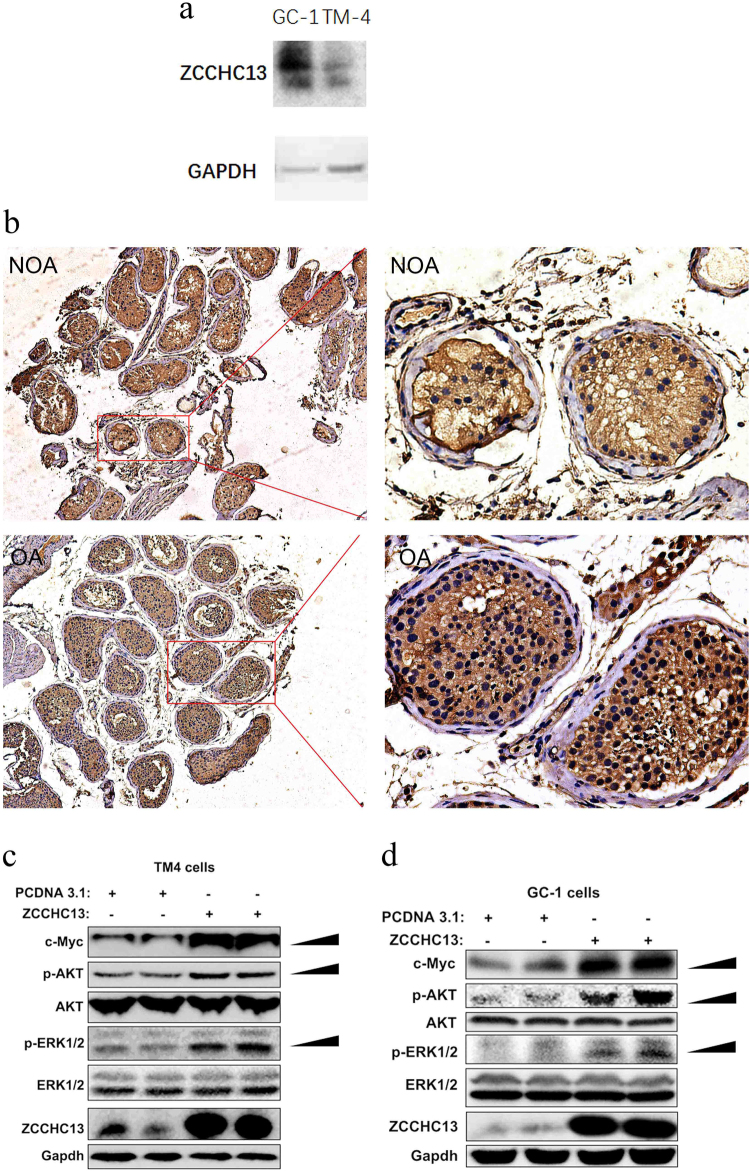
Testicular distribution and molecular function of ZCCHC13. **a** Immunoblotting analysis were employed to assess the expression level of ZCCHC13 in GC-1 and TM4 cells. GAPDH serves as a loading control. **b** Immunohistochemistry demonstrating ZCCHC13 protein presented in germ cells from testis biopsy from man with OA controls (normal spermatogenesis), and had significantly decreased expressions of ZCCHC13 protein in man with NOA at low power 100× and high power 400×. **c** TM4 cells and **d** GC-1 cells show that the expression levels of c-MYC, p-AKT, and p-ERK were remarkably increased following ZCCHC13 overexpression. GAPDH served as a loading control

As shown in Fig. 6a, immunoblot analysis demonstrated that the TM4 Sertoli cell line expressed a relatively low level of ZCCHC13 compared with GC-1 cells. This result might suggest a more important role for the ZCCHC13 protein in GC-1 cells (corresponding to a stage between the type B spermatogonia and the primary spermatocytes). The Sertoli cell is the only somatic type inside seminiferous tubules and secretes diverse functional glycoproteins and peptides that lead to normal germ cell development and maturation^22^. To further investigate the role of ZCCHC13 in human spermatogenesis, we assessed ZCCHC13 expression by immunohistochemistry in testicular tissues from men with NOA and men with OA. Immunohistochemistry indicated high levels of ZCCHC13 expression within the nucleus of germ cells (spermatogonia, spermatocytes, spermatids, and spermatozoa) of OAs compared with NOAs (Fig. 6b). We proposed that ZCCHC13 is implicated in vital biological processes and an aberrant methylation of the ZCCHC13 promoter could be one possible cause of idiopathic NOA.

### ZCCHC13 activates AKT/MAPK/c-MYC pathway in male germ cells

Our integrated analysis identified a potentially interesting novel gene, ZCCHC13, that is regulated by DNA methylation mechanisms. However, the biological role of ZCCHC13 has not yet been reported. The transcription factor c-MYC plays an essential role in cell cycle regulation and proliferation^23^. The AKT and MAPK signaling pathways regulate c-MYC expression and promote c-MYC stability^24^. Thus, we wondered whether these growth-enhancing molecules would be activated in the male germ cells with a concomitant increase in the ZCCHC13 protein, which might reflect the molecular function of ZCCH13 for NOA. Therefore, the phosphorylated protein levels of AKT and ERK were measured along with the protein levels of c-MYC in TM4 cells and GC-1 cells. We found that the activation status of these molecules was significantly increased in the ZCCHC13 overexpressed group relative to the empty vector controls (Fig. 6c, d), suggesting that basic growth-enhancing pathways are activated by the expression of constitutive ZCCHC13 in male germ cells, which likely functions to promote sperm proliferation.

### Increased *ZCCH13* expression by 5-Aza is mediated through DNA demethylation

**Fig. 7:**
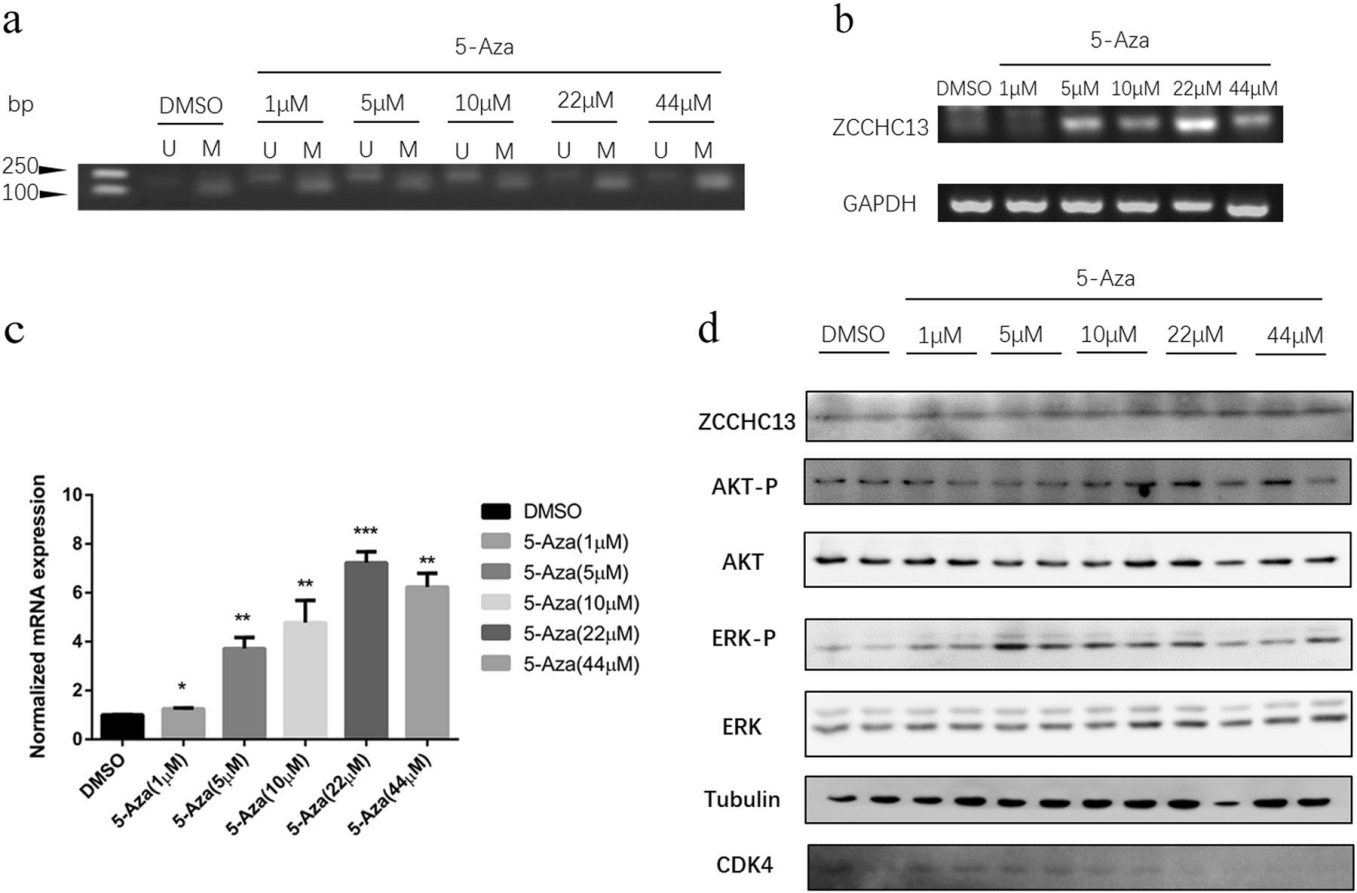
Increased expression of *ZCCHC13* in response to 5-Aza treatment. **a** MSP analyses showed that all the five different concentrations of 5-Aza can attenuate the DNA methylation within the promoter of *ZCCHC13* in a dose-dependent manner in GC-1 cells. *U* unmethylated MSP amplicons; *M* methylated MSP amplicons. **b** RT-PCR analysis of *ZCCHC13* expression in 5-Aza-treated GC-1 cells. **c** Quantitative PCR analysis in terms of ZCCHC13 expression levels were normalized to those of the internal control GAPDH. Values are the mean ± S.D. (error bars) of at least three determinations. Significant differences (*p < 0.05, **p < 0.01) between the control (DMSO) and experimental groups are marked with asterisks. **d** GC-1 cells were treated with various doses of 5-Aza for 48 h. The protein levels of ZCCHC13, p-AKT1, AKT1, p-ERK1/2, ERK, and CDK4 measured by Western blotting analysis

We also examined whether 5-Aza increased *ZCCHC13* expression through DNA demethylation within the promoter region of *ZCCHC13*. We have established an MSP method that readily determines the methylated or unmethylated alleles of the *ZCCHC13* promoter. MSP assays showed that GC-1 cells had a methylated promoter, and an alteration in the methylation status of the *ZCCHC13* promoter was closely correlated with different concentrations of 5-Aza (Fig. 7a). To confirm the effect of 5-Aza on mRNA expression of *ZCCHC13*, reverse transcription-PCR (RT-PCR) and real-time quantitative PCR were performed in GC-1 cells after a 24 h treatment with different concentrations of 5-Aza. As shown in Fig. 7b, c, 5-Aza treatment resulted in a dose-dependent increase of *ZCCHC13* mRNA expression with significant effects at 1, 5, 10, and 22 µM (*p* < 0.01). Although slightly reduced mRNA expression of *ZCCHC13* was found after a 44 µM 5-Aza treatment in GC-1 cells, the increase and differences were still statistically significant compared with controls (*p* < 0.01). Moreover, immunoblotting indicated that 5-Aza increased protein expression of ZCCHC13 in a dose-dependent manner (Fig. 7d). This increase was accompanied by enhanced phosphorylation of AKT1 (Ser129) and ERK1/2 (Thr202/Tyr204), especially at a 10 µm concentration of 5-Aza. Taken together, our results indicate that the 5-Aza-induced increase in ZCCHC13 expression is associated with demethylation within the *ZCCHC13* promoter region.

### 5-Aza-induced GC-1 cell proliferation inhibition caused by CDK4 retardation

**Fig. 8:**
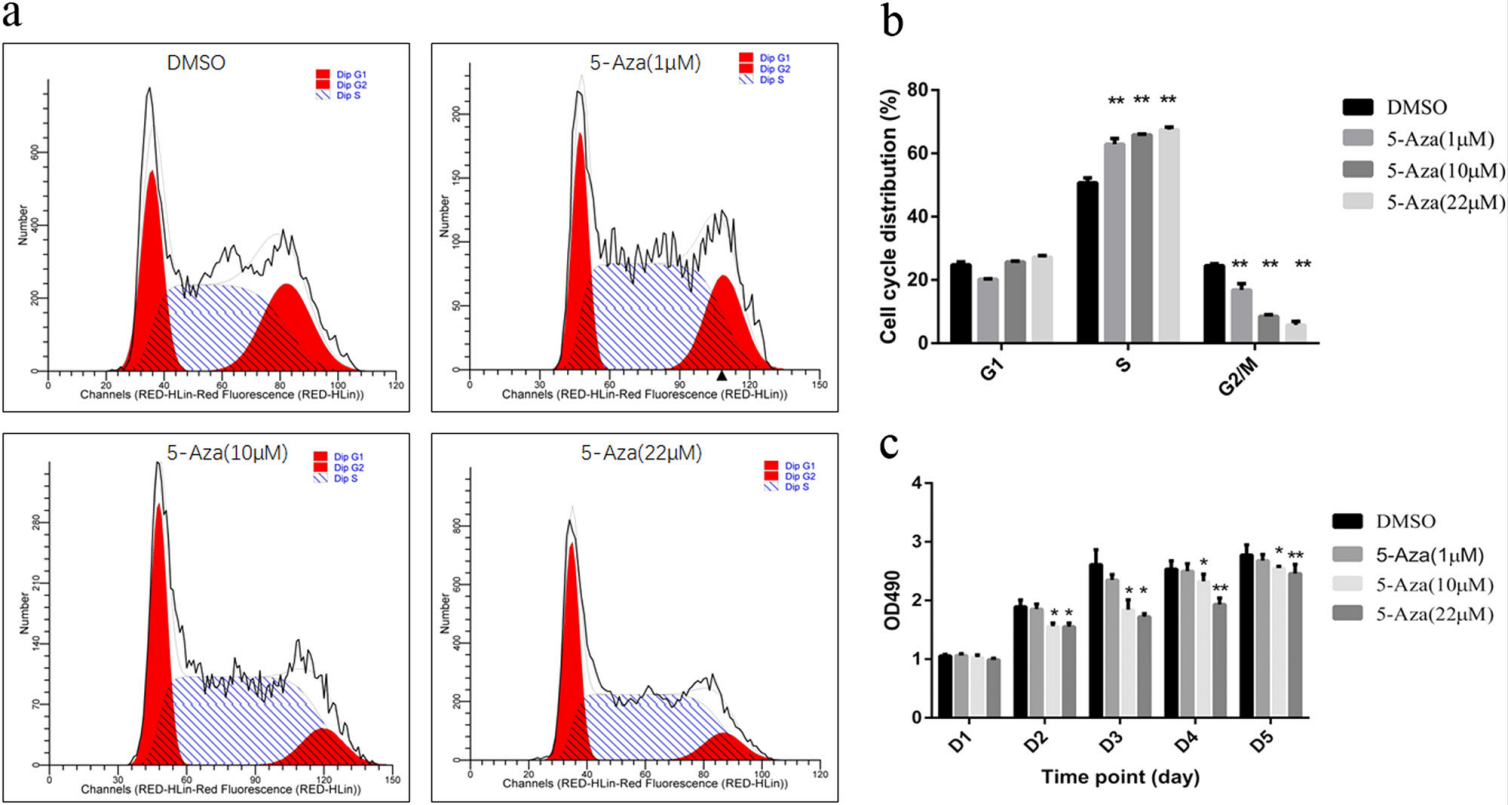
5-Aza suppressed the GC-1 cells growth. **a** Flow cytometric analysis of cell cycle in GC-1 cells treated with 5-Aza at the indicated doses for 24 h. Cells were analyzed by flow cytometry after staining with PI. **b** Quantization of flow cytometry data according to cell cycle phase for the groups indicated. **c** 5-Aza significantly inhibited cell proliferation in a dose-dependent and time-dependent manner. The cell viability was determined by MTS assay (*n* = 3). Data represent the mean ± SD. Significant differences (*p < 0.05, **p < 0.01) between the control (DMSO) and experimental groups are marked with asterisks

Unexpectedly, our results demonstrated the ability of 5-Aza to down-regulate CDK4 expression in a dose-dependent manner (Fig. 7d). Despite some evidence for the substantial toxicity of 5-Aza to male reproduction, only a few studies have been published regarding the effects of 5-Aza and its mechanism in normal germ cells. Therefore, we tested cell growth status under 5-Aza treatment in normal reproductive cells by using GC-1 cells (a mouse testicular spermatogonia cell line). To determine the effect of 5-Aza on the cell cycle distribution in GC-1 cells, cells were incubated with four different concentrations of 5-Aza (1, 5, 10, and 22 µM). Using FACS analysis, we found that demethylation induced by 5-Aza resulted in significantly fewer cells in the G2/M phase of the cell cycle and a distinct increase of S-phase GC-1 cells compared with the control group (Fig. 8a, b), which supported the abnormal changes in the G2/M transition of cell cycle genes in the NOA microarray analysis. To confirm the cell cycle-suppressive properties of 5-Aza in GC-1 cells, we tested whether 5-Aza also caused inhibitory effects over time. As shown in Fig. 8c, 5-Aza significantly inhibited GC-1 cell growth, as evidenced by the MTS assay, in a dose-dependent and time-dependent manner. The growth of 10% of GC-1 cells was inhibited by 10 or 22 µM 5-Aza treatment for 24 h compared with control cells, and the difference was statistically significant (*p* < 0.05).

## Discussion

The association between abnormal DNA methylation and spermatogenesis failure has been reported, but whether these aberrant DNA methylation profiles cause azoospermia and how they affect spermatogenesis failure are still unknown. To the best of our knowledge, our study constitutes the first attempt to integrate genome-wide DNA methylation and gene expression data from testicular tissue samples to identify genes that are possibly regulated by DNA methylation for NOA. DNA methylation is a type of epigenetic modification that can effectively promote gene silencing. Our study focuses on genes with a negative correlation between DNA methylation and gene expression and highlights the statistically significant difference between NOA and OA groups. A total of 2,597 genes were analyzed due to the negative correlation between methylation and expression. These genes may be useful for developing biomarkers for diagnostic or prognostic purposes. From a biological standpoint, GO, KEGG, and PPI were applied to identify the top changed genes and their functional categories. In addition, we established a pipeline for investigating genes that were dysregulated by DNA methylation for complex diseases when using primary samples. The application of integrative approaches not only increases the robustness of internal validation but also reduces potential confounding effects from distinct resources^11^.

To explore the functional interpretation of our results and provide some new clues for a follow-up mechanism study, we characterized the events of potential functional significance of genes for which DNA methylation changes were significantly inversely correlated with changes in expression. Functional analysis identified two significant biological processes and pathways: one is involved in the G2/M transition of cell cycle and the other involves the MAPK signaling pathway. Further study showed that the demethylating agent 5-Aza induced spermatogonia GC-1 cells to increase the percentage of cells in the G0/G1 phase and decrease the percentage of cells in the G2/S phase. The inhibitory effect of 5-Aza on GC-1 cell growth according to the MTS assay also confirmed the cell cycle results. More importantly, we observed significantly decreased expression of CDK4 after treatment of 5-Aza. A previous study had reported that atrophic seminiferous tubules within the testes of *Cdk4*-null mice showed reduced numbers of spermatogonia and spermatocytes, suggesting an essential role for CDK4 in germ cell development^25^. The present data provided a molecular mechanism of 5-Aza-induced damaged effects in testicular cells, which can be employed for the selection of possible preventive measures.

**Fig. 9:**
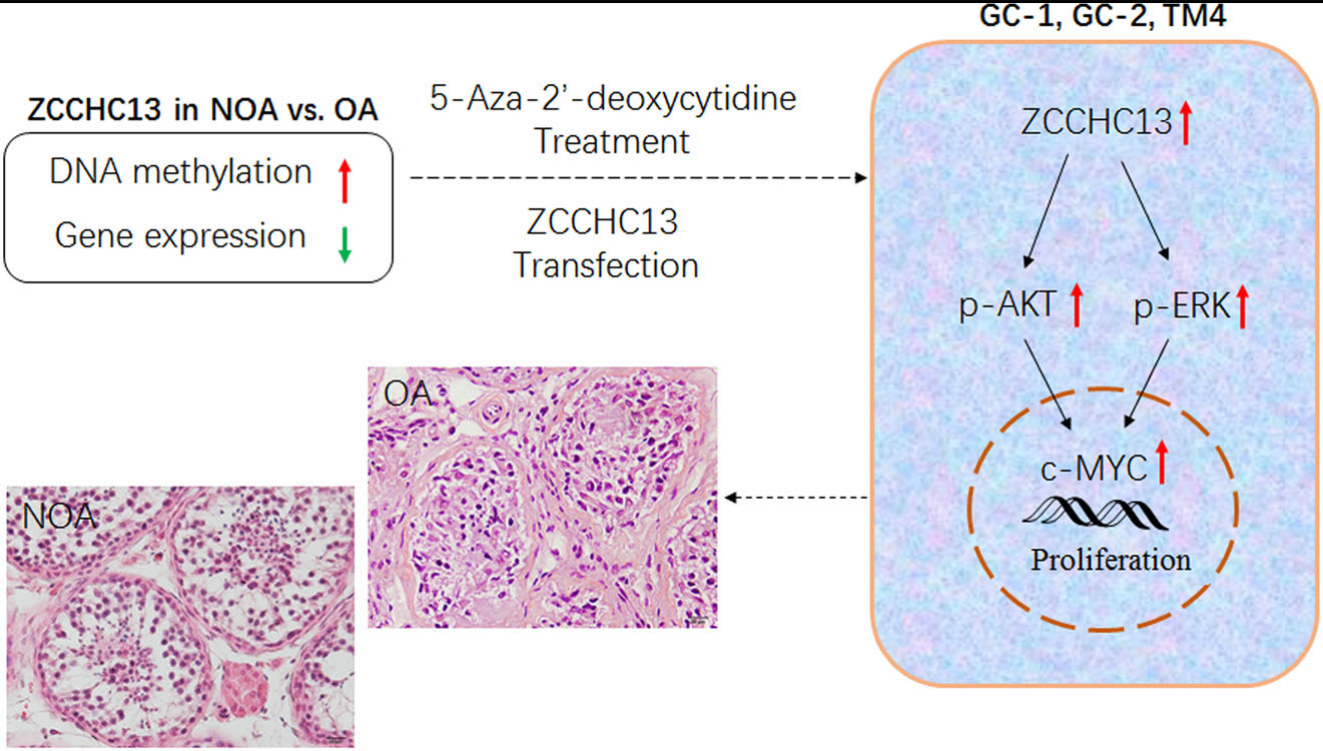
Proposed model elucidating the role of *ZCCHC13* in male germ cells. ZCCHC13 activates p-AKT and p-ERK signaling, which stimulated c-MYC activation. c-MYC increases cell cycle regulatory protein expression through transcription of target genes. *ZCCHC13* was identified as coordinately down-regulated and hypermethylated in NOA patients with meiotic arrest. We hypothesized that ZCCHC13 regulates germ cell proliferation via c-MYC-dependent PI3K/Akt and MAPK pathway

*ZCCHC13* was previously identified as a novel imprinted gene in a well-known epigenetic regulatory mechanism region termed the X-chromosome inactivation enter^26^, but the biologic significance of *ZCCHC13* is still unknown. Our data demonstrated for the first time that ZCCHC13 expression stimulated AKT/MAPK/c-MYC signaling in mouse germ cell lines, which is an important step toward understanding the role of *ZCCHC13* in spermatogenesis. It is notable to find that 5-Aza significantly increased *ZCCHC13* expression in GC-1 cells. *ZCCHC13* is a member of a family of zinc-finger CCHC-type containing DNA/RNA-binding proteins that are involved in mRNA transcription and protein translation^27^. One of the best examples is the Nanos protein, which contains a conserved zinc-finger domain that includes two consecutive CCHC-type zinc-finger motifs and contributes to germ cell development in mice^28^. In this study, we characterized the effect of methylation and expression of *ZCCHC13* in men with NOA. Moreover, we demonstrated the presence of ZCCHC13 protein in OA with normal spermatogenesis and a lower expression of ZCCHC13 protein in NOA. From the microarray results of integrative analysis, we demonstrated that the phenomenon of hypermethylation of the CpG site within the *ZCCHC13* promoter leads to decreased mRNA and protein expression in NOAs. Altered *ZCCHC13* gene expression may therefore be a novel marker for men with idiopathic NOA. As summarized in our working model shown in Fig. 9, the role of ZCCHC13 in spermatogenesis involves the promotion of c-MYC expression and activation of phosphorylation of AKT, ERK1/2. Hypermethylation of *ZCCHC13* in NOA patients leads to reduction of c-MYC expression, which influences cell proliferation and differentiation by transcription and activation of downstream target genes.

## Conclusions

Our study constitutes the first attempt to integrate DNA methylation and expression data from testicular tissue samples in the context of NOA. Of the methylated genes with inversely correlated expression, we identified a novel biomarker, ZCCHC13, that is highly expressed within the nucleus of germ cells of testis in men with normal spermatogenesis, but decreased in men with NOA. Our data indicated that ZCCHC13 expression is positively correlated with the AKT/MAPK/c-MYC levels and is regulated by methylation modification. The molecular function and epigenetic regulation of ZCCHC13 expression in germ cells will enhance our understanding of current knowledge on abnormal methylation-derived male infertility.

## Materials and methods

### Patients

Testicular biopsy specimens were obtained from four patients (aged 28–29 years) with NOA and from three patients (aged 25–28 years) with OA for microarray analysis. These patients underwent testicular sperm extraction (TESE) for assisted reproduction and/or diagnostic biopsy for histological examination. For the histological evaluation, the specimens were stained with hematoxylin and eosin (H&E) and analyzed by microscopy. Each patient was first diagnosed with azoospermia due to the lack of ejaculated spermatozoa in a semen examination. OA was defined as in Okada et al.^29^, and: (1) motile spermatozoa were obtained from a microsurgical epididymal sperm aspiration or (2) a large number of mature spermatozoa were provided via TESE. The ideal controls in the study are a normal male population with known fertility; however, difficulties in sampling testicular tissues made this strategy impractical. Instead, control samples consisted of males who had no history of meiotic impairment or infertility and exhibited normal spermatogenesis upon histological examination. Karyotype analysis and Y-chromosome microdeletion analysis were performed on all patients to confirm a normal karyotype. Additionally, none of the controls experienced adjuvant hormonal treatment prior to orchiectomy. Infertile male patients who visited Xiamen University Affiliated First Hospital had a routine semen examination on the basis of the 2010 WHO criteria. The ethics committees of Xiamen University Affiliated First Hospital (Institutional Review Board Number: KYX-2015-001) approved the study design. All subjects provided written informed consent.

### RNA extraction and gene expression microarray

Testicular biopsies were stored at −80 °C immediately after surgery until further RNA purification. Tissues were homogenized with a homogenizer, and total RNA was purified from the testicular samples using the miRNeasy Micro Kit (catalog no. 217084, Qiagen, Germany) according to the manufacturer’s instructions. Then, on-column DNase digestion and in-solution DNase digestion were performed to remove DNA contamination. The concentration, purity, and integrity of the purified RNA was determined by a UV spectrophotometer NanoDrop ND-1000 (Peqlab, Erlangen, Germany) and the Agilent 2100 Bioanalyzer (Agilent Technologies, USA).

Gene expression microarray was performed as previously described^30–32^. Briefly, Cy3-labeled cRNA for microarray hybridization was prepared with the One-Color Low Input Quick Amp Labeling Kit (Agilent, USA) and then purified using the RNeasy Mini Kit (Qiagen, Germany). Cy3-labeled cRNA was fragmented and hybridized to the array for 17 h at 65 °C in a rotating Agilent hybridization oven. After hybridization, arrays were washed and dried, then scanned immediately on the Agilent Microarray Scanner. Intensity values of each scanned slide were extracted using Agilent Feature Extraction software (version 10.7.3.1; Agilent Technologies).

### Gene expression analysis

Raw data analyses were performed with GeneSpring GX software (Version 12.0; Agilent Technologies). The intensity values were log 2 transformed by quantile normalization^33^. The Welch *t*test (*p*values) was applied to identify differentially expressed genes in NOA compared to OA. The *p* values were corrected by the false discovery rate of Benjamini and Hochberg (*q* values) analyses. Fold change (FC) values were calculated for each gene as the difference between the mean intensity of the NOA samples and mean intensity of the OA samples. Genes with an FC value >2 or <1/2 and a *q*value <0.05 were considered to be differentially expressed. Quality control analysis of microarray gene expression data was performed as previously described^34^.

### DNA isolation, preparation, and DNA methylation microarray

Testicular tissues were dissolved in 200 μl of lysis buffer from the DNA Micro Kit (catalog no. 56304, Qiagen, Germany) and incubated with proteinase K overnight at 56 °C for two nights. DNA was extracted according to the manufacturer’s protocol (QIAamp DNA Micro Kit, Qiagen), and the DNA concentration was determined at 260 nm using a NanoDrop ND-1000 spectrophotometer (Nanodrop Technologies Inc., Wilmington, NC, USA). Bisulfite modification of 500 ng of DNA from each sample was performed with the EZ DNA Methylation Kit (Zymo Research, Orange, CA, USA).

Infinium HumanMethylation450 BeadChips (Illumina Inc.) were applied to analyze the DNA methylation profiles. Bisulfite-treated DNA (4 µl) from each sample was prepared for hybridization on the microarray. The signal intensities were extracted with the GenomeStudio software (version 2011.1; Illumina). The methylation level of each CpG site was recorded as a *β*value, indicating the ratio of the methylated signal intensity over the sum of the methylated and unmethylated intensities at each locus. The average *β* value represents a methylation signal that ranged from 0 (completely unmethylated) to 1 (completely methylated).

### DNA methylation analysis

The bioconductor R package minfi was used to pre-process the data and for quality control. The Wilcoxon's rank test was conducted between NOA and OA samples. Probes were considered to be statistically significant and differentially methylated if the adjusted *p* value was <0.05 and the Δ*β* was >0.2 or <0.2. For this, we calculated the Δ*β* value as the difference in the average *β*values between the NOA and OA samples.

### Combining gene expression and DNA methylation profile**s**

To combine methylation and expression data, we established a two-step analysis strategy. First, significantly differentially methylated genes between NOA and OA patients were analyzed. Genes that were differentially methylated in NOA compared with OA were selected if the absolute value of Δ*β* was >0.2 and *p*value was <0.05. Second, for each differentially methylated gene, the Spearman's rank correlation for the median of the methylation level of CpGs in an amplicon against the expression probes was calculated using the function cor.test in R. Significantly negative correlations were considered if the correlation coefficient *ρ* was <0 and *p*value was <0.05, and significantly positive correlations were considered if *ρ* was >0 and *p*value was <0.05. Differentially expressed genes that matched methylated genes were then studied.

In addition, we conducted unsupervised clustering analysis, which demonstrated the separation of NOA and OA samples with evidence of gene clusters that were differentially expressed or methylated. Hierarchical clustering was performed using Ward linkage with Euclidean distance for samples. A clustering method available in the Charm package in R was applied to the differentially methylated CpGs^35^.

### GO and KEGG analysis

To find the biological mechanisms underlying the genes analyzed above, we conducted GO term and KEGG pathway analysis using Cytoscape V2.7 (http://cytoscape.org/) with the ClueGo V1.3 plug-in^36^, which is able to extract biological features and annotations of anti-correlated genes. To only obtain significant enrichment categories of the GO terms and KEGG pathways, the *p*value was adjusted with right-sided hypergeometric tests and corrected by Benjamini–Hochberg adjustment (*q* value). The categories with *q* value <0.05 were selected for further analysis, which were considered to be significantly deviated from the expected distribution. The results were visualized with ClueGO to generate clusters of functions associated with genes.

### PPI network construction and analysis

The latest experimentally confirmed human protein–protein interaction (PPI) data are available from the human PPI database (http://www.hprd.org/), which has been widely applied in human PPI network research for various diseases. NetworkAnalyzer^37^ (http://www.mpi-inf.mpg.de/) was used to analyze the topological properties of biological networks, describing networks as collections of nodes and edges. Cytoscape software was used for network visualization.

### Cell culture and drug treatments

GC-1 cells (mouse spermatogonia cell line) and TM4 cells (mouse Sertoli cell line) were maintained in Dulbecco’s modified Eagle’s medium (DMEM) supplemented with 10% fetal bovine serum in 5% CO_2_ incubators at 37 °C. Cells were incubated with various doses (1, 10, 22, and 44 µM) of 5-Aza (Sigma, St. Louis, MO, USA) for the duration of the experiment.

### ZCCHC13 plasmid construction and transfection

The *ZCCHC13* coding region was amplified by PCR by using the following primers: 5′-CGGAATTCATGAGCAGTAAGGATTTCTTCGC-3′ and 5′-GGGGTACCGCCTGGGACATTCCTTGGCTAGAT-3′. The ZCCHC13 open reading frame was confirmed by sequencing and cloned into eukaryotic expression vector pcDNA 3.1 (Invitrogen) using the restriction sites *Eco*RI and *Kpn*I to create pcDNA 3.1/ZCCHC13. Plasmid transfections were performed using the liposome reagent Fugene 6 (Roche Molecular Biochemicals) according to the manufacturer’s instructions.

### Cell cycle assay

GC-1 cells were cultured in DMEM with 10% fetal bovine serum and were seeded onto 12-well plates the day before 5-Aza treatment. Twenty-four hours after treatment, cells were harvested and fixed with 70% alcohol in phosphate-buffered saline (PBS) at 4 °C. After washing in PBS, cells were treated with 1% RNase (w/v) at 37 °C for 30 min. Cells were then stained with propidium iodide (PI) (250 µg/µl) at room temperature for 10 min and analyzed with a Flow cytometer (Guava Technologies, Burlingame, CA, USA). The percentages of cells in the G0/G1 phase, S phase, and G2/M phase were evaluated by the ModFit software (BD, Franklin, NJ, USA).

### Cell proliferation assay

GC-1 cells were seeded in triplicate into 96-well plates (1,500 cells per well) before drug treatment. After overnight recovery, the cells were treated continuously with freshly prepared 5-Aza from day 1 to day 5. Cell proliferation at different time points was measured using a CellTiter 96^®^ Aqueous One Solution Cell Proliferation Assay (MTS; Promega, Madison, WI, USA), and absorbance (OD 490 nm) was measured with a 96-well plate Microplate reader MK3 (Thermo Fisher Scientific Corporation, Waltham, MA, USA).

### Western blot analysis

GC-1 cells were treated with 5-Aza. TM4 cells and GC-1 cells were transfected with pcDNA 3.1/ZCCHC13. Modified cells were washed with ice-cold Dulbecco’s PBS and then lysed for 30 min on ice in lysis buffer containing protease inhibitor cocktail tablets (Roche, Nutley, NJ, USA). The protein concentration was determined using a BCA Protein Assay Kit (Pierce, Rockford, IL, USA). Equal amounts of protein (40 µg) were separated on a 12% sodium dodecyl sulfate-polyacrylamide gel electrophoresis gel under reducing conditions, after which they were electrophoretically transferred onto PVDF membranes. The blots were then probed with primary antibodies: antibody anti-AKT (1:500), anti-p(Ser129)-AKT1 (1:500), anti-ERK1/2 (1:500), anti-p(Thr202/Tyr204))-ERK1/2 (1:500), anti-CDK4 (1:500), anti-GAPDH (1:1,000), anti-ZCCHC13 (1:400), and anti-c-MYC (1:1,000). All primary antibodies were purchased from Sangon Biotech (Shanghai, China), except for anti-ZCCHC13 antibody (Abcam, ab104509) and anti-c-MYC antibody (Abcam, ab32072). Anti-rabbit or anti-mouse horseradish peroxidase (HRP)-conjugated IgG (Santa Cruz Biotechnology) was then used as appropriate for secondary antibodies at a 1:5,000 dilution; the blots were subsequently developed using enhanced chemiluminescence reagents (Amersham Pharmacia Biotech, Piscataway, NJ, USA).

### Methylation-specific PCR

DNA from GC-1 cell lines was extracted using a DNeasy System (Qiagen) according to the manufacturer’s instructions. DNA (0.5 µg) was converted with sodium bisulfite using an EZ DNA Methylation Kit (Zymo Research), and modified DNA was amplified by PCR. The sequences of the methylation-specific primers were 5′-TAAAGATTGTAAGGATTTTAAACGA-3′ (forward) and 5′-ATCGATAACACTTAACCTAAACGC-3′ (reverse); the sequences of the unmethylation-specific primers were 5′-TTAAAGATTGTAAGGATTTTAAATGA-3′ (forward) and 5′-ATCAATAACACTTAACCTAAACACA-3′ (reverse), corresponding to the *ZCCHC13* promoter region. Methylation-specific PCR (MSP) was performed in a total volume of 25 µl. PCR reactions for both primer sets contained 100 ng of bisulfate-modified DNA, 1.5 mM MgCl_2_, 0.25 mM dNTPs, 0.5 μM each primer, and 1.25 U of HotStar Taq DNA polymerase (Qiagen). The PCR program consisted of a denaturing step of 10 min at 95 °C followed by 35 cycles of 30 s at 95 °C, 30 s at 50 °C annealing temperature (M and U), and 30 s at 72 °C, with a final extension of 10 min at 72 °C. Methylated and unmethylated DNA amplified products were detected on 1.2% agarose gels.

### Quantitative real-time RT-PCR analysis

Total RNA of GC-1 cells was isolated using TRizol reagent according to the manufacturer’s guidelines (Invitrogen). Total RNA (2 µg) was reverse transcribed into cDNAs using the commercial Quanti-Tect Reverse Transcription Kit (Qiagen) according to the manufacturer’s protocol. A portion (2 μl) of each cDNA sample was amplified with the TransStar Top Green qPCR Super Mix (TransGen, Beijing, China) using the Agilent Mx3005P qPCR system. Glyceraldehyde 3-phosphate dehydrogenase (GAPDH) was selected as the internal control. The primers were 5′-AACGAGAGAGACGCCAACAC-3′ (forward) and 5′-CGCATCGGTAACACTTGACCT-3′ (reverse) for *ZCCHC13*. The primers were 5′-AGGTTGTCTCCTGCGACTTCA-3′ (forward) and 5′-GGGTGGTCCAGGGTTTCTTAC-3′ (reverse) for GAPDH. The relative standard curve (2−ΔΔCt) method was used to determine relative mRNA expression.

### Immunohistochemistry

Immunohistochemical analysis was performed on formalin-fixed, paraffin-embedded tissue sections. Samples were then heated in a boiling water bath for antigen retrieval (10 mmol/L citrate buffer, pH 6.0, 20 min). The sections were allowed to cool in citrate buffer, washed three times with PBS, and incubated in blocking solution for 30 min. Next, samples were washed with PBS (three times, 10 min/time) and incubated overnight with primary rabbit polyclonal anti-ZCCHC13 antibody (1:100 dilution, Abcam, ab104509) at 4 °C. After being washed with PBS (three times, 10 min/time), the sections were incubated with HRP-conjugated goat anti-rabbit IgG for 1 h at room temperature. Finally, peroxidase activity was visualized using 0.05% diaminobenzidine (Sigma-Aldrich).

### Statistical analysis

For nonparametric data, multiple comparisons were made with the Kruskal–Wallis test, followed by Dunn’s multiple comparison test. For single comparisons, unpaired data were analyzed by Mann–Whitney *U* test and paired data were analyzed by Wilcoxon's matched pairs test. A *p* value <0.05 was considered statistically significant. Analyses were performed using GraphPad Prism version 5.00 for Windows (GraphPad Software, San Diego, CA, USA).
